# How Illness Context Shapes Caregiving: Comparative Ethnography of Diverse Caregiving Experiences

**DOI:** 10.1002/alz.085141

**Published:** 2025-01-09

**Authors:** Alma V Hernandez De Jesus, Brandi Ginn, Ignacia Arteaga Perez, Corey M Abramson, Daniel Dohan

**Affiliations:** ^1^ University of California, San Francisco, San Francisco, CA USA; ^2^ University of Arizona, Tucson, AZ USA

## Abstract

**Background:**

Prior research shows that caregiving for people living with dementia (PLWD) varies with cultural, institutional, and social structural context, but less is known about the role of context in dementias of different etiologies. We compared experiences of caregiving in frontal‐temporal dementia (FTD) versus non‐FTD dementias using community‐based comparative ethnography. We expected to find differences in caring for people living with FTD (PLWFTD) versus people living with other dementias (PLWOD). Our analytical intent was to show how cultural, institutional, and structural context shaped these experiences and to identify opportunities for intervention.

**Method:**

Data were obtained from three years of community‐based ethnography in diverse communities in five cities via participant‐observation (n = 282 daily fieldnotes) and in‐depth semi‐structured interviews (n = 80 PLWD and caregivers). For data collection, a diverse research team trained in observation and interview methods and met weekly to review field experiences. For analysis, we used a qualitative computational approach that included large language models to identify data about illness trajectory and caregiving experiences. We read through the resulting data deductively to examine our expected findings about PLWFTD versus PLWOD, and we used inductive analysis to make sense of unexpected and counterfactual findings. Our results highlight linkages among caregiving experiences, disease‐etiology, and social context.

**Result:**

As expected, we found differences in caregiving experiences of PLWFTD and PLWOD, and we also found many similarities. Among PLWFTD, earlier onset and more difficulty obtaining diagnosis created difficulties and delays in assuming a caregiving role. Following diagnosis, caregivers could find it challenging to recognize and manage their own response to the behavioral and personality changes associated with FTD. These compounded challenges experienced by all caregivers such as feelings of guilt, frustration over behavioral changes, structural barriers to care, and institutional challenges showed much‐needed respite.

**Conclusion:**

Caregiving for PLWD is taxing on multiple levels with some additional challenges related to dementia diagnosis. These findings contribute an in‐depth account of similarities and differences in caregiving experiences and can inform evidence‐based recommendations to support caregiving initiatives and PLWD. Among modifiable factors, our results suggest a need for mechanisms to support differential diagnosis and for provision of respite care.

